# Na^+^/K^+^ ATPase activity promotes invasion of endocrine resistant breast cancer cells

**DOI:** 10.1371/journal.pone.0193779

**Published:** 2018-03-28

**Authors:** Maitham A. Khajah, Princy M. Mathew, Yunus A. Luqmani

**Affiliations:** Faculty of Pharmacy, Kuwait University, Safat, Kuwait; Wayne State University, UNITED STATES

## Abstract

**Background:**

The Na^+^/K^+^-ATPase (NKP) is an important ion transporter also involved in signal transduction. Its expression profile is altered in various tumours including that of the breast. We studied the effect of inhibiting NKP activity in non-tumorigenic breast cell line and in estrogen receptor positive and negative breast cancer cells.

**Methods:**

Expression and localization of NKP and downstream signaling molecules were determined by RT-PCR, western blotting and immunofluorescence. Cell proliferation, apoptosis and cell cycle stage were determined using MTT, annexin V and flow cytometry. Cell motility and invasion were determined using wound healing and matrigel assays. Total matrix metalloproteinase (MMP) was determined by a fluorescence-based assay.

**Results:**

NKP was mainly localized on the cell membrane. Its baseline expression and activity were enhanced in breast cancer compared to the non-tumorigenic breast cell line. Ouabain and 3,4,5,6-tetrahydroxyxanthone (TTX) treatment significantly inhibited NKP activity, which significantly reduced cell proliferation, motility, invasion and pH-induced membrane blebbing. EGF stimulation induced internalization of NKP from the cell membrane to the cytoplasm. Ouabain inhibited EGF-induced phosphorylation of Rac/cdc42, profillin, ERK1/2 and P70S6K.

**Conclusions:**

The NKP may offer a novel therapeutic target in breast cancer patients who have developed metastasis, aiming to improve therapeutic outcomes and enhance survival rate.

## Introduction

The ion transporter sodium/potassium (Na^+^/K^+^)-ATPase pump (NKP) is located on the plasma membrane and is responsible for the regulation of ion homeostasis by exporting 3 Na^+^ in exchange for 2 K^+^. Four α, three β and one γ-subunit of NKP have been described [1]. The α-subunit is considered to be the catalytic part and contains the Na^+^, K^+^, Mg^2+^, ATP and ouabain (chemical inhibitor of the NKP activity) binding sites. The β-subunit is involved in the transport of the α-subunit to the plasma membrane as well as in the structural and functional maturation of the holoenzyme [2]. The γ-subunit is thought to be involved in the modulation of pump activity [3]. Other evidence suggests the involvement of NKP in signal transduction [4, 5] through activation of the protein kinase cascade [6] while inhibiting Src activity through direct interaction [7, 8]. NKP also modulates the activity of various signaling molecules important for cancer pathogenesis such as epidermal growth factor receptor (EGFR), mitogen-activated protein kinase (MAPK) and the PI3K/Akt/mTOR pathway [9, 10].

The NKP is expressed in various cells of non-cancerous origin such as neurons and cardiomyocytes. Altered expression level/activity of the pump has been reported in diabetes [11], hypertension [12], Alzheimer`s disease [13] and in various tumors including glioblastoma, non-small cell lung carcinoma, melanoma, colorectal carcinoma, bladder and breast cancer [14–19]. A recent study analyzed microarray data of breast cancer expression profiling and demonstrated a significant (1.5 fold) increase in the expression of the ATP1A1*gene* (coding the α1-subunit of NKP) in tissues obtained from different breast cancer patient groups (triple negative, Her2-positive, and Luminal A and B) compared to normal breast tissues [20]. A 2-fold reduction in the *ATP1A2* expression and lack of changes of expression in *ATP1A3* were also observed [20]. Although, as previously mentioned in regard to increased expression of the alpha subunit of the pump, other reports have demonstrated reduced NKP activity in breast cancer cells which were paralleled by cellular transition from epithelial to mesenchymal phenotype (EMT) in part due to reduced expression of tight junction (TJ) proteins [21]. Several lines of evidence suggest an important role of NKP in regulating cell-cell and cell-substrate interactions in addition to cell adhesion in both normal and cancerous cells [22]. This pump is also involved in the formation of TJ proteins needed for maintaining cell polarity [21] through regulating MAPK activity, and the re-distribution of TJ molecules such as ZO-1 and occludins. Furthermore, this pump is involved in translocation of the oncogene β-catenin from sub-membrane scaffold to the nucleus [23].

In this laboratory, we have established several endocrine resistant breast cancer cell lines by siRNA mediated knockdown of the estrogen receptor (ER) in MCF-7 cells. All of these lines with *acquired* resistance exhibit an EMT phenotype with enhanced expression of mesenchymal markers (such as vimentin), reduced expression of epithelial markers (such as E-cadherin), enhanced proliferation and motility and invasion towards various chemotactic agents including epidermal growth factor (EGF) [24–27]. We have recently reported that brief exposure of the ER-ve breast cancer cells to alkaline (but not acidic) pH extracellular environment induces morphological changes where the cells become rounded and shrink in size and form actin-rich bleb-like structures on the outer membrane. This also results in enhanced invasive potential towards serum components and EGF, in part due to enhanced MMP2/9 activity. Treatment with inhibitors of NKP prevented these morphological and functional changes associated with exposure to alkaline pH, which may offer a novel therapeutic target in breast cancer treatment [26, 27].

In this study, we examined the effect of NKP [by using chemical inhibitors ouabain and 3,4,5,6-tetrahydroxyxanthone (TTX), a synthetic xanthone derivative and siRNA-mediated knockdown] on various signaling molecules and cellular functions (proliferation, apoptosis, motility and invasion) in both *de novo* and *acquired* endocrine resistant breast cancer cell lines, and compared this with that of the ER+ve cell lines and the non-tumorigenic breast cell line MCF10A. We show here that NKP was mainly localized on the plasma membrane of all the tested cell lines, while its baseline expression level and activity was enhanced in breast cancer compared to the non-tumorigenic breast cell line. Ouabain and TTX treatment (1–10 μM) demonstrated equivalent inhibitory efficacy of NKP in pII cells. In addition, ouabain treatment (and to a lesser extent TTX treatment) significantly inhibited cell proliferation, motility and invasion. This was confirmed by using the siRNA-mediated knockdown of NKP in the highly proliferative and invasive ER–ve pII breast cancer cell line. The anti-proliferative effect of ouabain was in part transmitted through modulation of cell cycle and apoptotic machinery. Furthermore, NKP was expressed on the outer membrane of the newly formed blebs of pII cells upon exposure to alkaline pH environment. Ouabain pre-treatment inhibited these morphological changes, confirming previous findings, in part through reducing the expression of accessory molecules involved in the actin cytoskeletal machinery such as rac/cdc42 and profillin. EGF stimulation induced internalization of NKP from the cell membrane to the cytoplasmic compartment and inhibited EGF-induced phosphorylation of key signaling molecules such as ERK1/2 and P70S6K (the downstream target of mTOR pathway).

## Results

### Effect of estrogen/tamoxifen treatment on the tested cell lines

**Fig 1:**
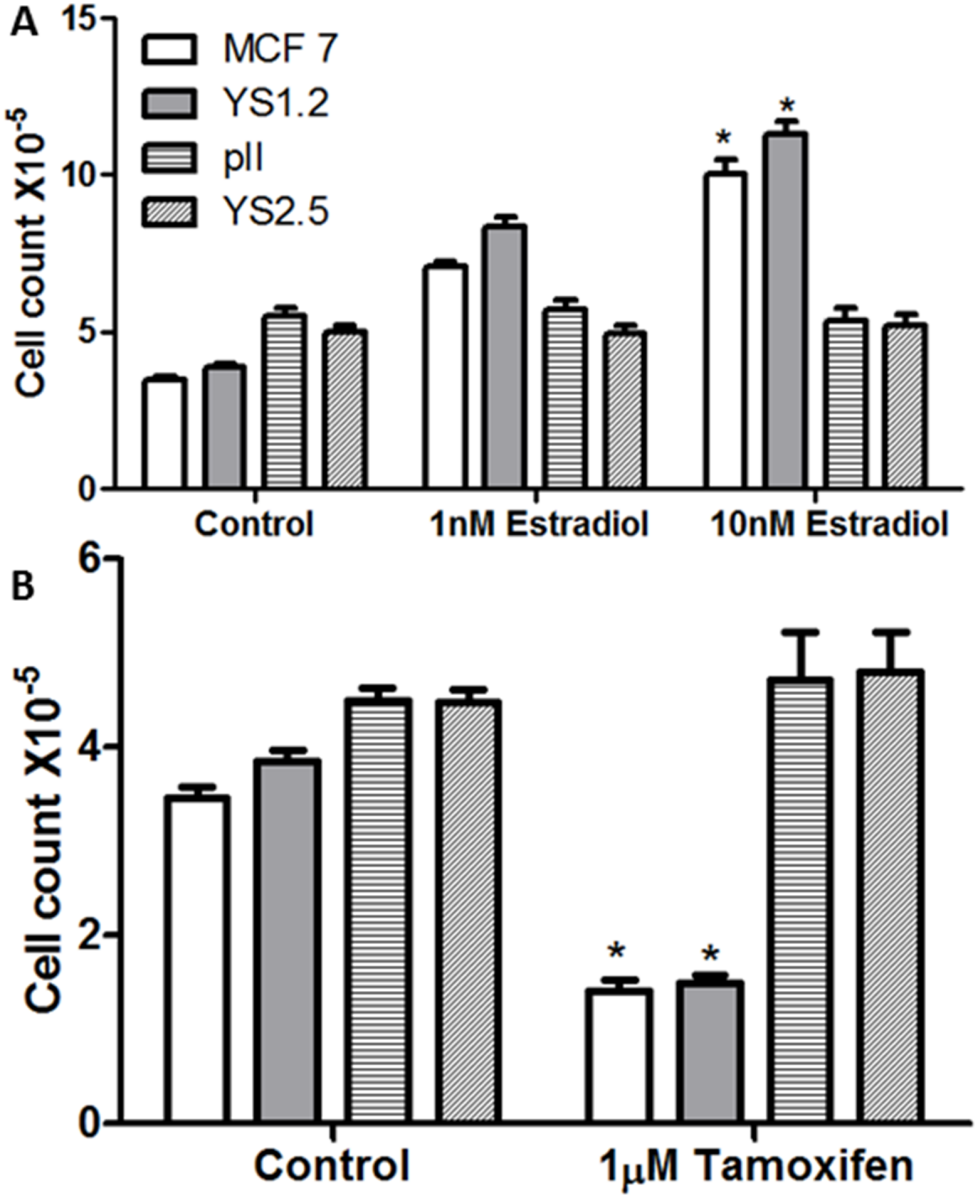
Effect of estradiol/tamoxifen treatment on cell proliferation. Approximately 10^4^ cells were seeded into microwell plates and allowed to grow over 4 days in the presence of vehicle (control, open bars) or various concentrations of estradiol or tamoxifen. Cells were harvested and growth was determined by the MTT assay. Histobars represent means ± SEM of at least 3 independent determinations. Asterisk denotes significant difference from control with p < 0.05.

MMT assay was performed for the tested cell lines to confirm their sensitivity/resistance to estrogen/tamoxifen treatment respectively. In ER + breast cancer cell lines (MCF-7 and YS1.2), estradiol treatment (100 nM for 4 days) significantly increased cells proliferation, while tamoxifen treatment (1 μM for 4 days) significantly inhibited their proliferation (Fig 1B). Neither treatments affected the proliferation rate of the ER–ve breast cancer cell lines (pII and YS2.5).

### NKP expression and activity in cell lines

**Fig 2:**
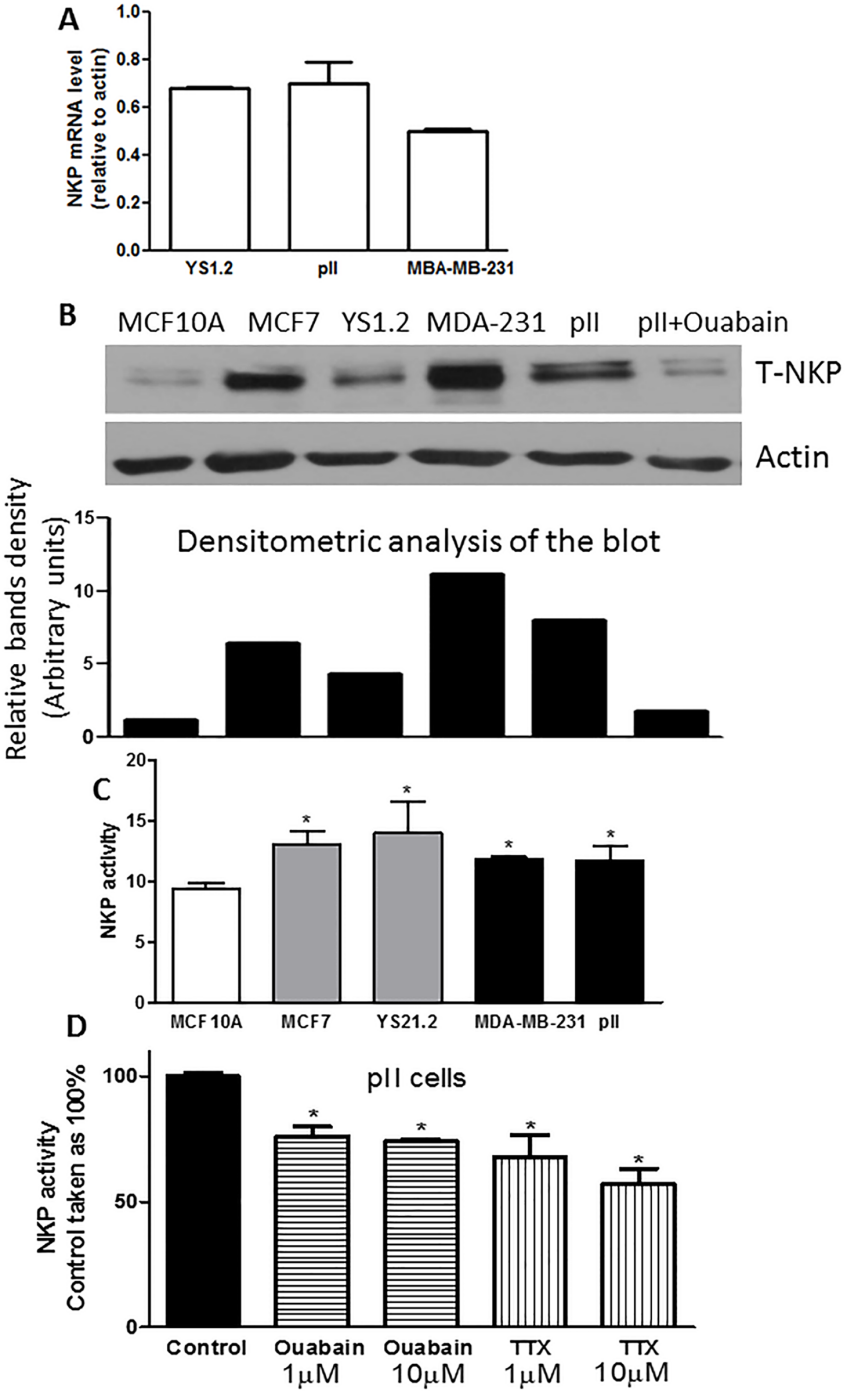
NKP expression and activity in normal and breast cancer cell lines. Panel A: NKP mRNA expression in ER+ (YS 1.2) and ER-ve (pII and MDA-MB-231) breast cancer cells. RNA was extracted from cells, converted to cDNA and PCR amplified. Ct values were converted to ratios relative to actin levels as described in Methods. Panel B: NKP protein expression level in the membranous compartment of the tested cell lines was determined by western blotting. Membranous fraction lysate protein (3 μg) was electrophoresed on 10% SDS polyacrylamide gel, blotted onto nitrocellulose membrane and probed with antisera to T-NKP and actin (loading control). This blot represents one of 3 independent determinations. NKP activity in the membranous fraction of all cell lines (panel C), and in pII cells in response to ouabain/TTX treatment (panel D) was determined by a colorimetric NKP activity kit. Histobars represent means ± SEM of at least 3 independent determinations. *denotes significant difference from MCF10A (panel C) or pII control (panel D) with p <0.05.

**Fig 3:**
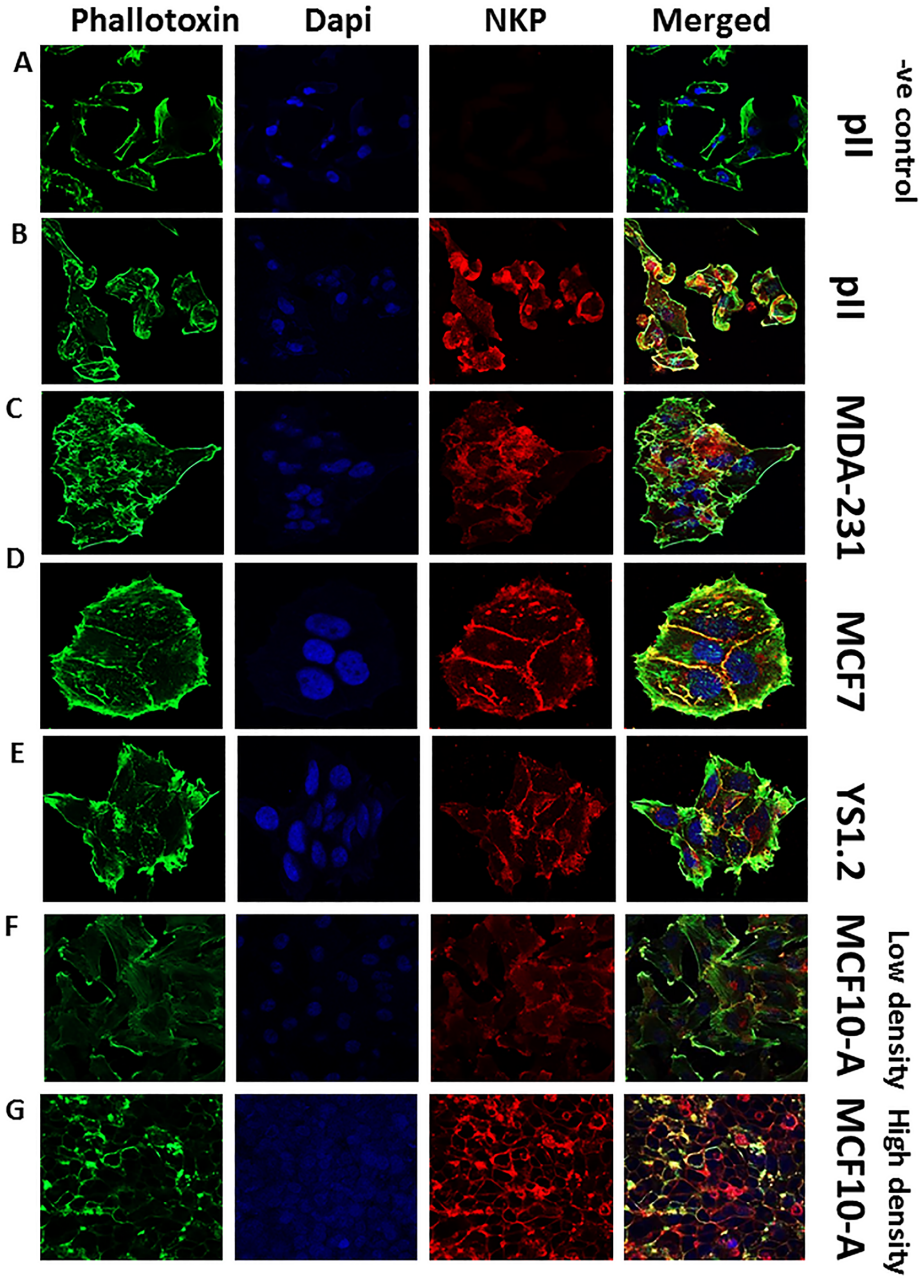
NKP localization in normal and breast cancer cell lines. Cells were seeded into 8-chambered slides and allowed to grow for 48 h at 37o C/ 5% CO2. Cells were then fixed and stained with NKP antibody (red), phallotoxin (green) and DAPI (blue). Panel A represents a negative control where pII cells were incubated with secondary antibody only.

No statistically significant difference was observed in the level of NKP mRNA between the ER+ and ER-ve breast cancer cell lines (Fig 2A). At the protein level, western blotting analysis of the membranous fraction of the cells indicated enhanced expression in cancer cells compared to the non-tumorigenic breast cell line (MCF10A), which was reduced by ouabain treatment (1 μM) in pII cells (Fig 2B). This also reflected enhanced baseline NKP activity of the membranous fraction of cancer cells compared to the non-tumorigenic breast cell line (Fig 2C). Ouabain and TTX treatment (1–10 μM) significantly reduced NKP activity in pII cells by 30–40% (Fig 1D). It should be noted that ouabain treatment to measure NKP expression/activity was over 24h and no cell death was observed at this early time point. Immunofluorescence analysis confirmed the membranous localization of NKP (Fig 3). It should be noted that the antibody used in western blotting analysis and the RT-PCR primers used in the study are specific to the alpha subunit of the Na-K-ATPase pump which is coded by the *ATP1A1* gene.

### Effect of ouabain/TTX on cell proliferation

**Fig 4:**
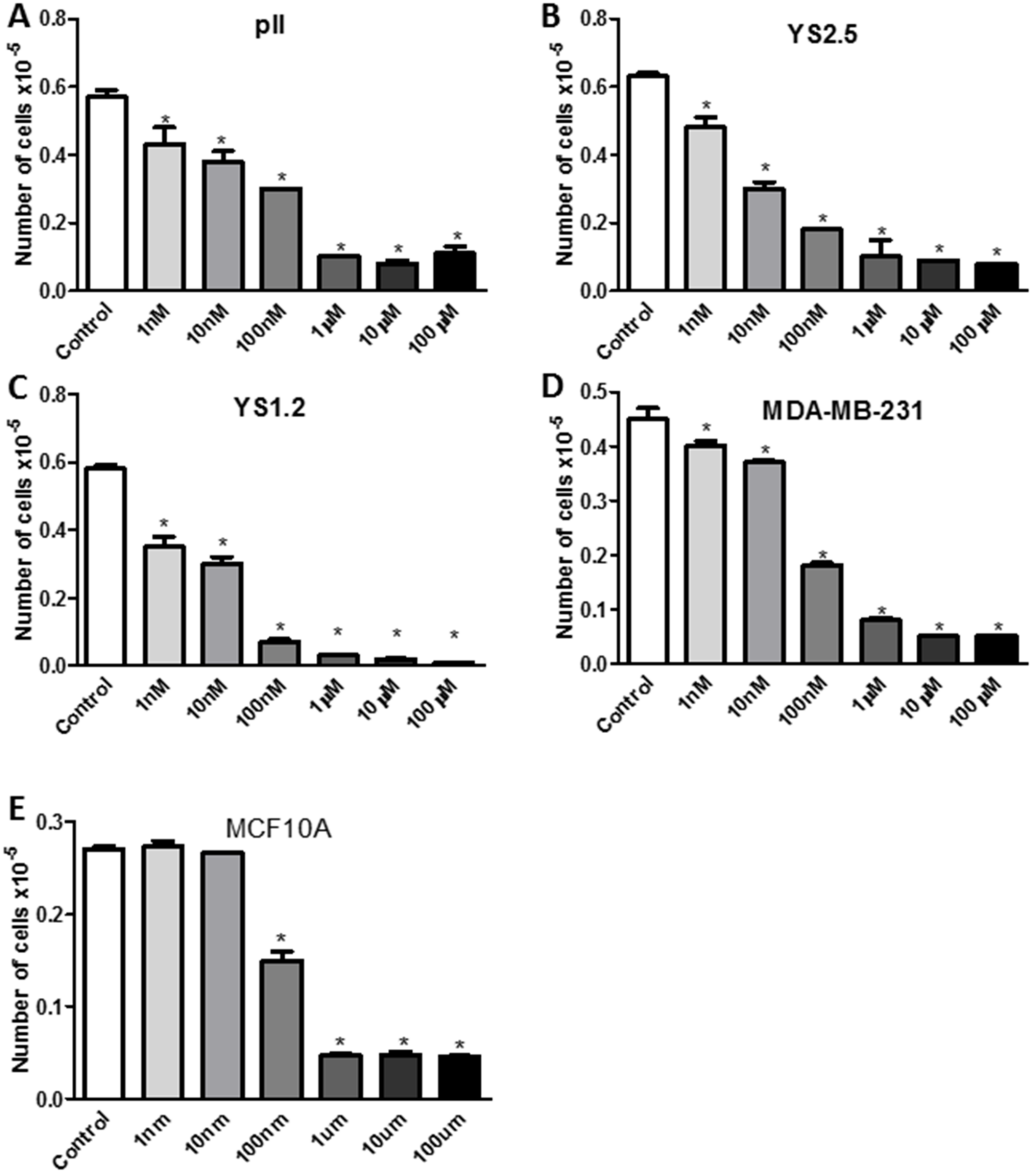
Effect of ouabain treatment on cell proliferation. Approximately 10^4^ cells were seeded into microwell plates and allowed to grow over 4 days in the presence of vehicle (control, open bars) or various concentrations (1 nM-100 μM) of ouabain. Cells were harvested and growth was determined by the MTT assay. Histobars represent means ± SEM of at least 3 independent determinations. Asterisk denotes significant difference from control with p < 0.05.

**Fig 5:**
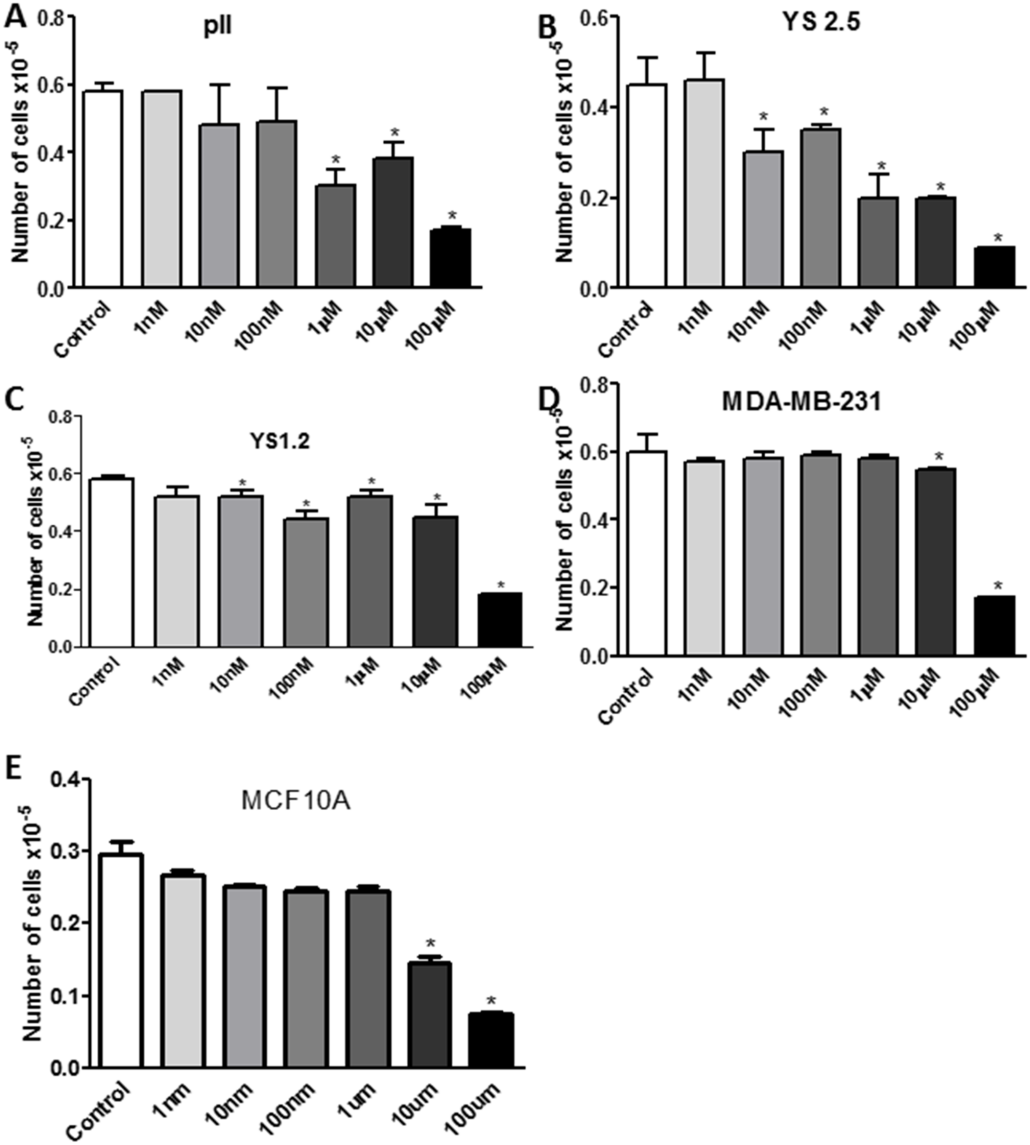
Effect of tetrahydroxyxanthone treatment on cell proliferation. Approximately 10^4^ cells were seeded into microwell plates and allowed to grow over 4 days in the presence of vehicle (control, open bars) or various concentrations (1 nM-100 μM) of tetrahydroxyxanthone. Cells were harvested and growth was determined by the MTT assay. Histobars represent means ± SEM of at least 3 independent determinations. Asterisk denotes significant difference from control with p < 0.05.

Figs 4 and 5 show results of MTT assay performed after 4 days of treatment with either vehicle (control) or increasing concentrations of ouabain or TTX in all cell lines. A preferential anti-proliferative effect of ouabain at 1 nM (although modest; 10–30%) was observed in breast cancer cells compared to the non-tumorigenic breast cell line MCF10A. Approximately 50–60% anti-proliferative effect was observed in all cell lines treated with ouabain at 10 nM without affecting the viability of MCF10A cells. This inhibitory effect was further enhanced to 60–90% using higher concentrations of ouabain (100 nM- 100 μM; Fig 4). The IC50 of ouabain was 26.4 nM for pII, 5.25 nM for YS2.5, 7 nM for YS1.2, 21.2 nM for MDA-MB-231, and 149 nM for MCF10A. Thus, the order of sensitivity of the cell lines to ouabain from the most to the least sensitive was YS2.5 > YS1.2 > MDA-MB-231 > pII > MCF10A.

TTX treatment was generally less effective than ouabain in inhibiting cell proliferation (Fig 5). A significant anti-proliferative effect (50–60%) of TTX was observed using concentrations from 1 μM and higher in all cell lines except MDA-MB-231 and MCF10A (TTX showed significant anti-proliferative effect against MDA-MB-231 and MCF10A cells at 10–100 μM; Fig 5D and 5E). The IC50 of TTX was 440 nM for pII, 130 nM for YS2.5, 400 nM for YS1.2, 9.5 μM for MDA-MB-231, and 10.4 μM for MCF10A. Thus, the order of sensitivity of the cell lines to TTX from the most to the least sensitive was YS2.5 > YS1.2 > pII > MDA-MB-231 > MCF10A.

**Fig 6:**
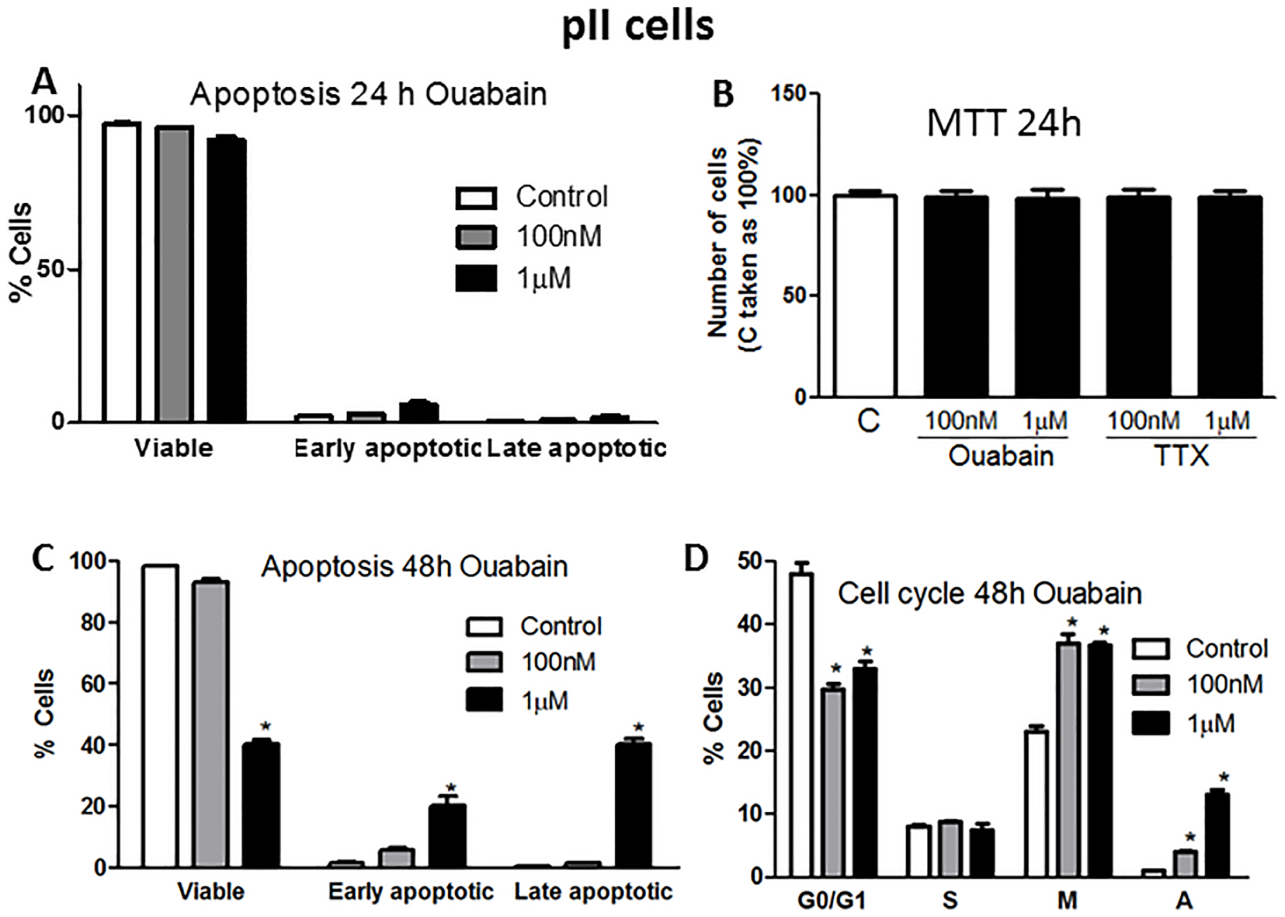
Effect of ouabain treatment on pII cell apoptosis and cell cycle. Approximately 10^4^ pII cells were seeded into a 6 well plate and allowed to attach overnight. Either vehicle (control, open bars) or ouabain (at 100 nM or 1 μM) was added. Samples were then analyzed by flow cytometry after 24 h of incubation for apoptosis (A) or proliferation (B), or after 48 h of incubation with the drug for apoptosis (C) and cell cycle phases (G0/G1; resting, S; synthesis, M; mitotic and A; apoptotic) (D). Histobars represent means ± SEM of at least 3 independent determinations. Asterisk denotes significant difference from control with p < 0.05.

We next determined if the anti-proliferative effect of ouabain in pII cells could be through modulation of cell apoptosis and/or the cell cycle machinery. Fig 6A and 6B shows that ouabain treatment for 24 h (which was subsequently used to measure cell motility and invasion) did not induce pII cell apoptosis or inhibited cell proliferation. Fig 6C shows a significant reduction in the % of viable cells and a significant increase in the % of apoptotic cells after 48 h of ouabain treatment (1 μM). Using the propidium iodide-based technique, ouabain treatment (100 nM-1 μM, 48 h) was found to significantly reduce the proportion of cells in the G0/G1 phase with a significant shift into the M and A phases (Fig 6D).

### Effect of NKP on breast cancer cell motility

**Fig 7:**
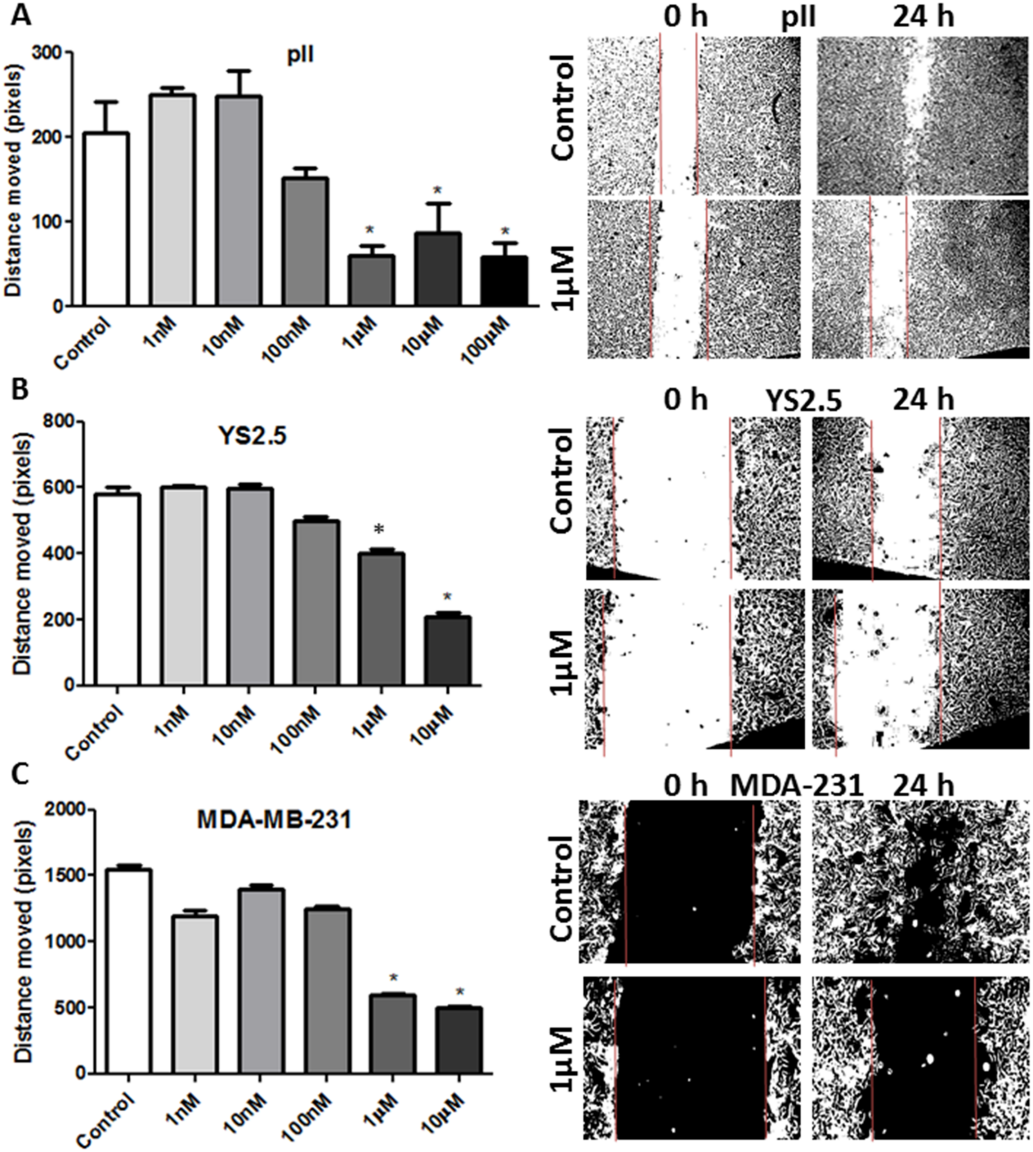
Effect of ouabain treatment on cell motility. The mean distance moved (in pixels) as a measurement of cell motility for all cell lines treated with vehicle (control, open bars) or various concentrations of ouabain (1 nM- 100 μM) determined after 24 h of incubation. Histobars represent means ± SEM of at least 3 independent determinations. Asterisk denotes significant difference from control with p < 0.05. The photographs in the right panels represent an example of motility for each cell line.

**Fig 8:**
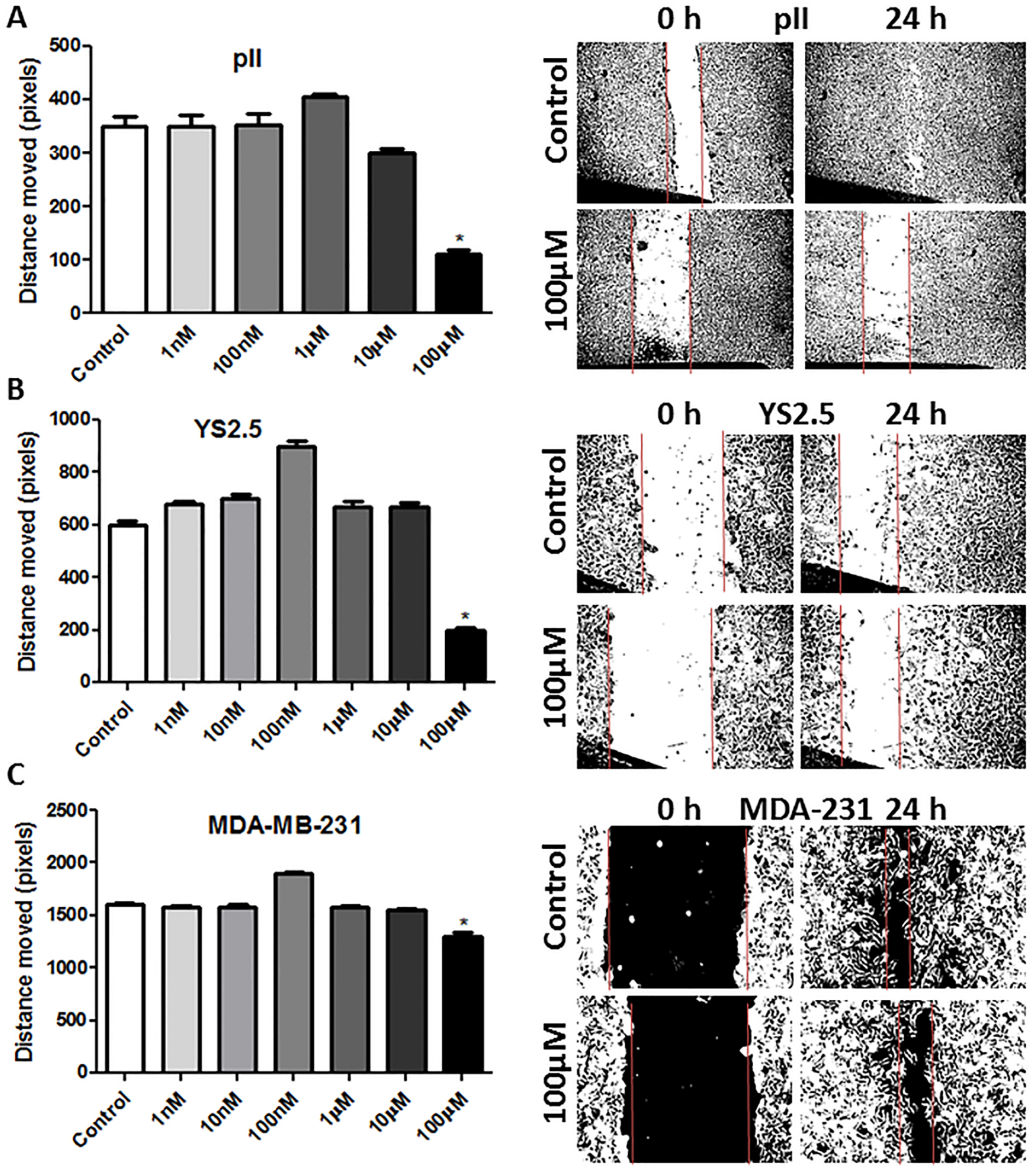
Effect of tetrahydroxyxanthone treatment on cell motility. The mean distance moved (in pixels) as a measurement of cell motility for all cell lines treated with vehicle (control, open bars) or various concentrations of tetrahydroxyxanthone (1 nM- 100 μM) after 24 h of incubation. Histobars represent means ± SEM of at least 3 independent determinations. Asterisk denotes significant difference from control with p < 0.05. The photographs in the right panels represent an example of motility for each cell line.

**Fig 9:**
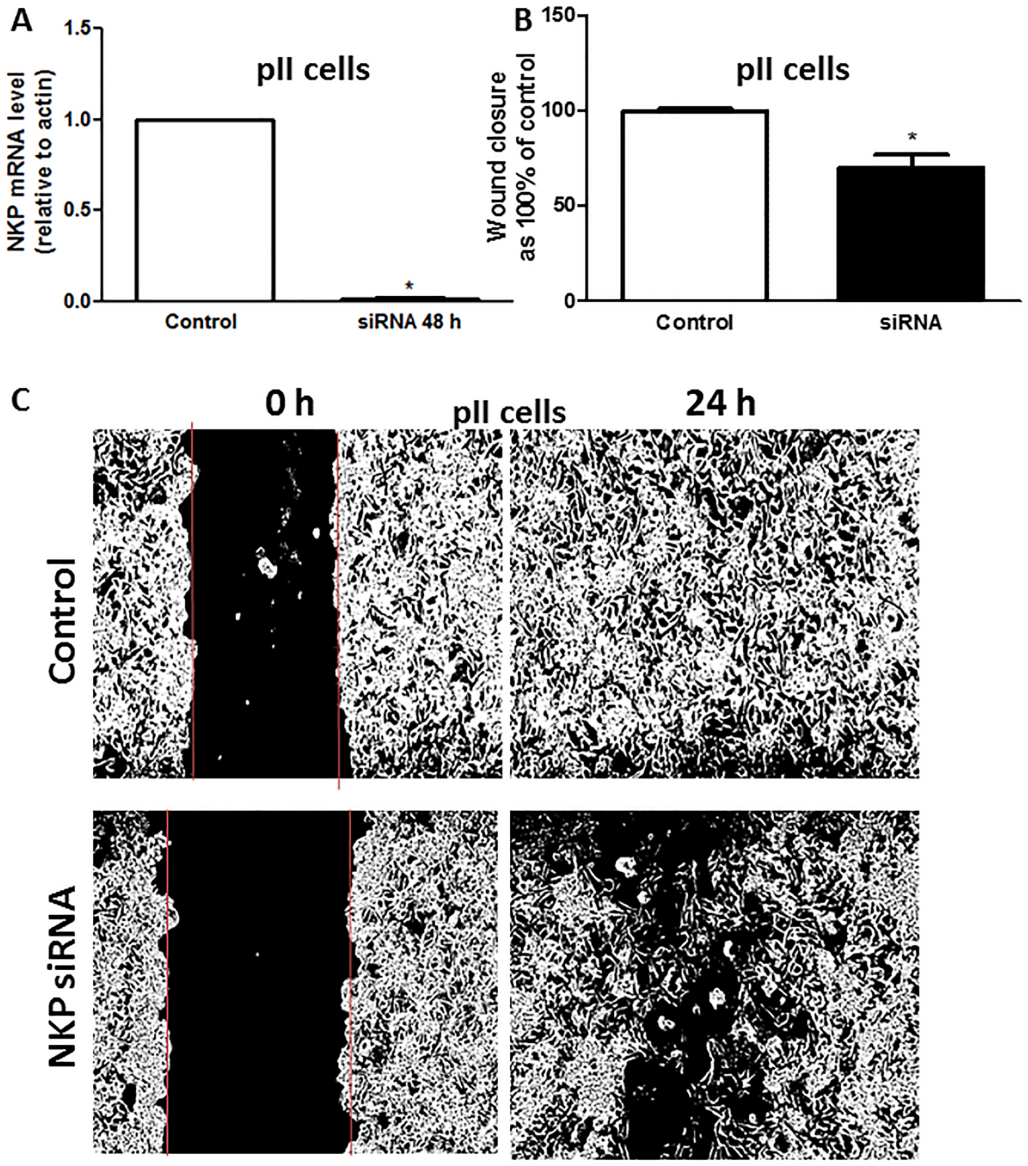
Effect of siRNA-mediated knockdown of NKP on pII cell motility. Panel A: NKP mRNA expression in pII cells transfected with a scrambled sequence (control, open bar) or NKP siRNA (48 h, solid bar). RNA was extracted from cells, converted to cDNA and amplified by PCR. Ct values were converted to ratios relative to actin. Panel B: mean distance moved (in pixels) as a measurement of cell motility for pII cell transfected with a scrambled sequence (control, open bar, taken as 100%) or NKP siRNA (48 h, solid bar) determined after 24 h of incubation. Histobars represent means ± SEM of at least 3 independent determinations. Asterisk denotes significant difference from control with p < 0.05. Panel C: The photographs represent an example of motility for each treatment condition.

Ouabain/TTX treatment (1 nM-100 μM) for 24h did not affect cell proliferation/apoptosis using the MTT/Annexin V assays (Fig 6A and 6B). Fig 7 shows the wound healing (scratch) assay for three ER-ve breast cancer lines after ouabain treatment for 24 h. A significant inhibition of cell motility (30–70%) was observed using 1–10 μM ouabain. TTX was less potent than ouabain in reducing cell motility, with significant inhibitory effect observed at TTX concentration of 10 μM (Fig 8). Using another approach, NKP activity was transiently inhibited in pII cells by siRNA-mediated knockdown of its alpha-1 subunit, with almost complete reduction in its expression (Fig 9A). A significant reduction in cell motility was observed in these NKP-down-regulated cells compared to cells transfected with a scrambled vector (control, Fig 9B and 9C).

### Effect of NKP on breast cancer cell invasion

**Fig 10:**
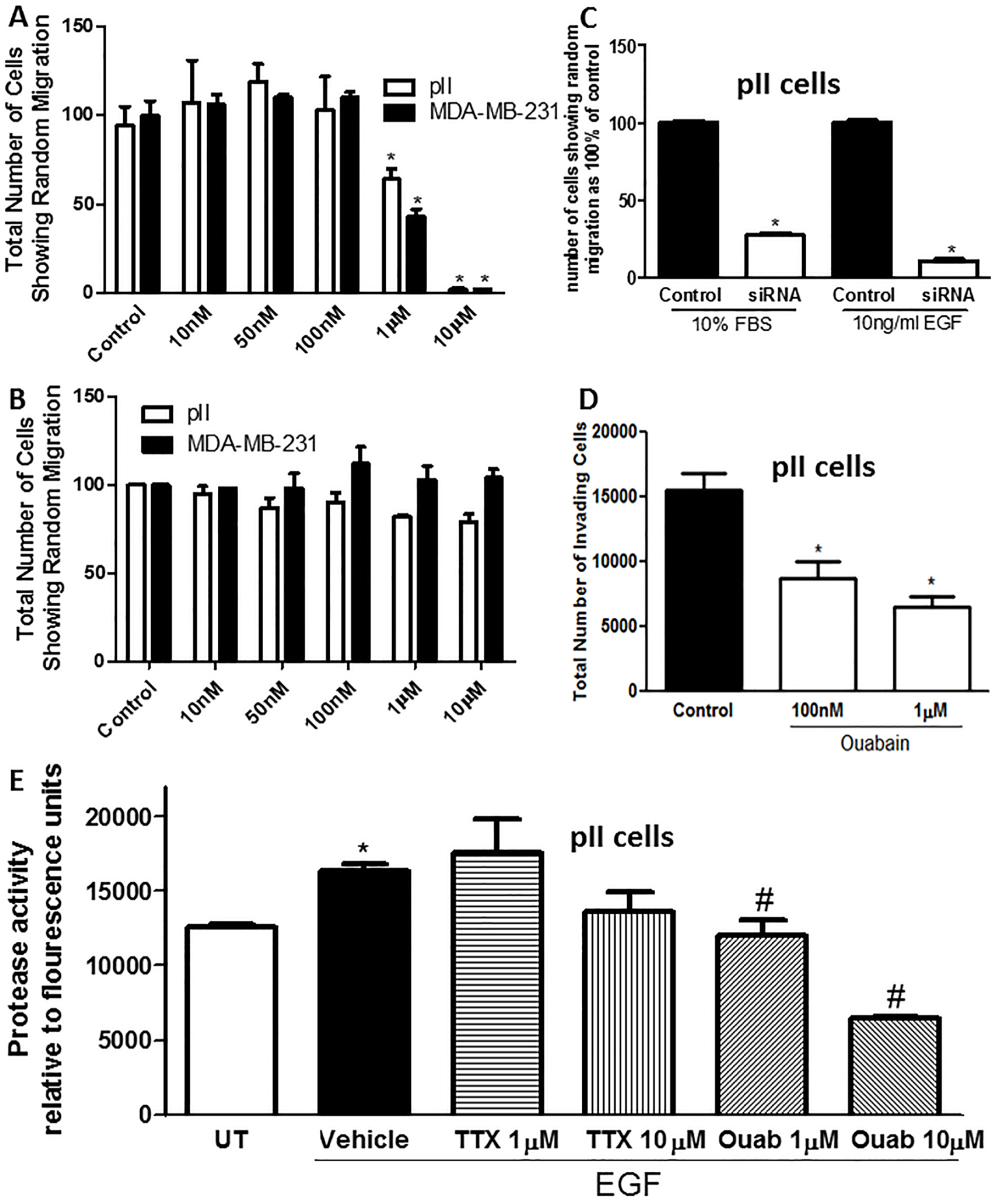
Effect of NKP inhibition on cell migration and invasion. The number of pII (open bars) or MDA-MB-231 (solid bars) cells showing migration through agarose towards serum components; either pre-treated with vehicle (control) or various concentrations (10 nM and 10 μM) of ouabain (A) or tetrahydroxyxanthone (B). Panel C: number of pII cells showing migration through agarose towards serum components (10% FBS) or EGF (10 ng/ml); either treated with scrambled sequence (control, solid bars, taken as 100%) or NKP siRNA (48 h, open bars). Panel D: number of pII cells showing invasion through the matrigel towards serum components present in the lower chamber; either pre-treated with vehicle (control, solid bar) or various concentrations (100 nM and 1 μM) of ouabain (open bars). Panel E: total MMP activity determined in pII cells in response to EGF stimulation and ouabain or TTX pre-treatment. Histobars represent means ± SEM of at least 3 independent determinations. Asterisk denotes significant difference from control/UT, # denotes significant difference from EGF/vehicle treated pII cells with p < 0.05.

Using the agarose migration assay, ouabain (but not TTX, Fig 10B) treatment (Fig 10A) significantly reduced pII and MDA-MB-231 cell migration towards serum components at concentrations of 1–10 μM with 40–90% inhibitory effect respectively. Also, siRNA-mediated knockdown of the NKP alpha 1 subunit in pII cells significantly (80–90%) reduced cell invasion towards serum components and EGF (Fig 10C). We also used the matrigel invasion assay to confirm the findings with the agarose assay, and indeed, ouabain treatment significantly reduced pII cell invasion through the basement membrane extract (BME) towards serum components (Fig 10D). Using a fluorescence-based assay to measure EGF-induced total MMP activity, we observed a significant reduction (40–60%) in the presence of ouabain (1–10 μM) (Fig 10E), but not TTX.

### Effect of NKP on alkaline pH-induced morphological changes

**Fig 11:**
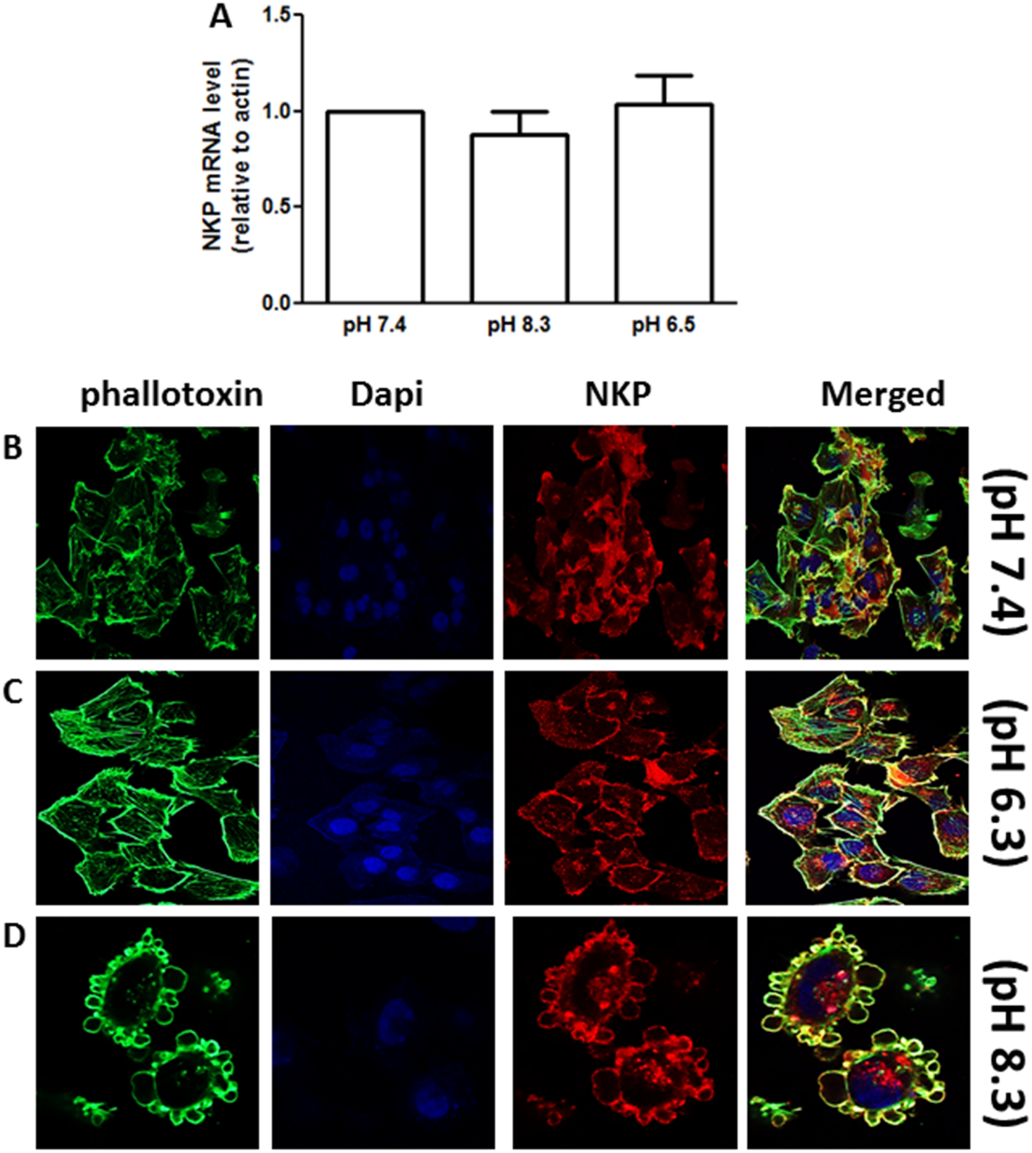
Effect of alkaline exposure on NKP expression and localization in pII cells. Panel A: NKP mRNA expression in pII cells exposed to various pH conditions (pH 7.4, 8.3, and 6.5 for 1 h). Histobars represent means ± SEM of at least 3 independent determinations. Panels B-D: Cells were seeded into 8-chambered slides and allowed to grow for 48 h at 37o C/ 5% CO2. Cells were exposed to various pH conditions (for 1 h) then fixed and stained with NKP antibody (red), phallotoxin (green) and DAPI (blue).

**Fig 12:**
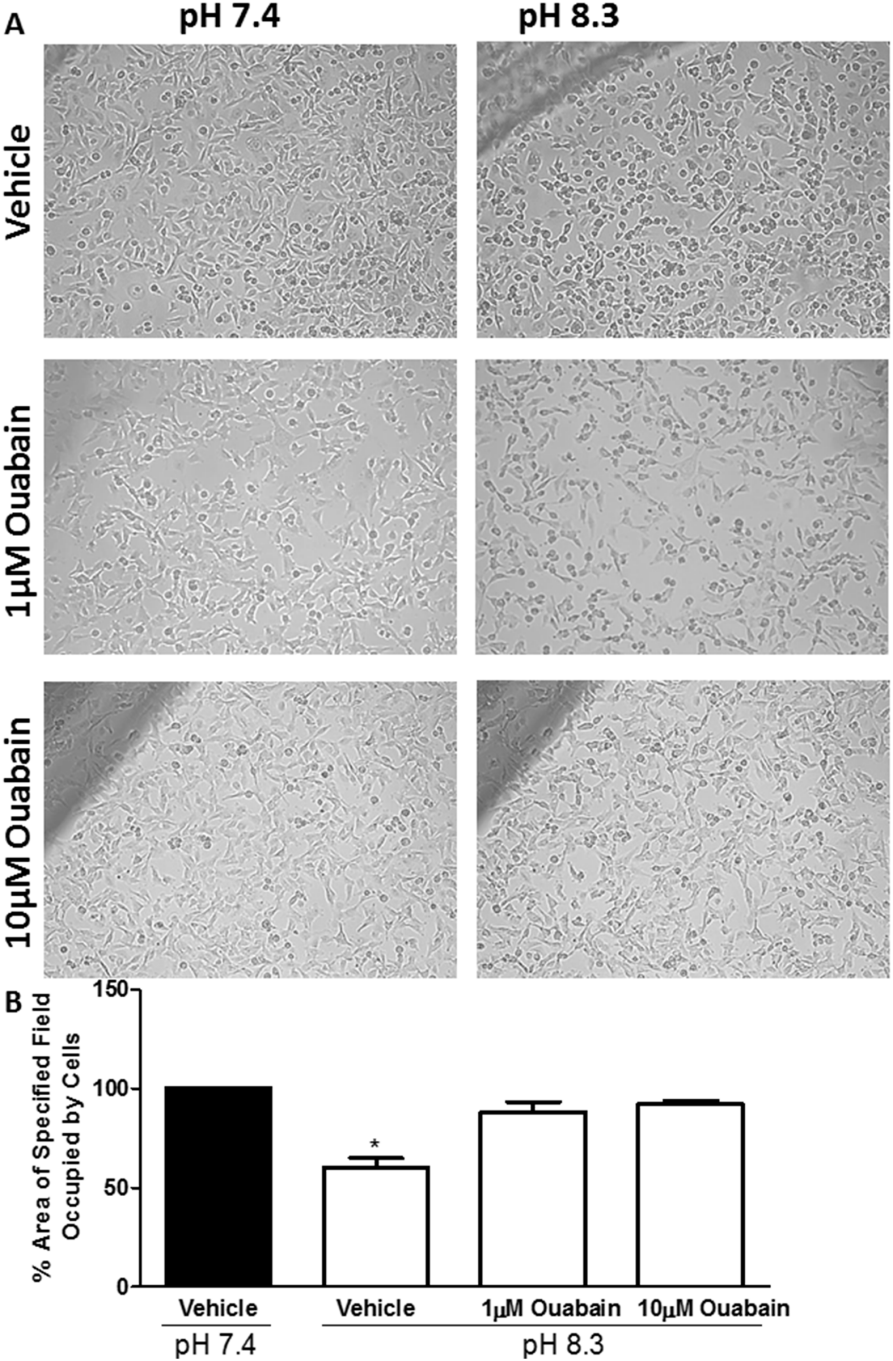
Effect of NKP inhibition on pH-induced morphological changes in pII cells. Panel A: Cells (either pre-treated for 1 h with vehicle or ouabain at 1 and 10 μM) photographed immediately after removal from the incubator (0 min, pH 7.4) and after 30 min of exposure to normal atmospheric conditions (as the pH increased to 8.3). Panel B shows quantitative analysis of at least 3 separate fields measured with Adobe Photoshop CS4 Measuring Tool from at least 3 independent determinations (means ± SEM). Asterisks denote significant difference from vehicle-treated cells at pH 7.4 (solid bar, set as 100%) with * p < 0.05.

We have previously observed that brief exposure of pII cells to alkaline (but not acidic) pH environment induces cell rounding and blebbing which correlates with enhanced cell invasion [26, 27]. Exposure of pII cells to either alkaline or acidic pH extracellular environment (1 h) did not modulate NKP levels (Fig 11A). As illustrated in Fig 11D, immunofluorescence staining of NKP shows its localization on the cell membrane of the newly formed blebs upon exposure to alkaline pH. No change in NKP localization was observed upon exposure to acidic pH (Fig 11C) compared to culturing the cells in neutral pH 7.4 (Fig 11B). In addition, pre-treatment (1 h) of pII cells with ouabain (1–10 μM) significantly inhibited cell rounding/blebbing upon subsequent exposure to alkaline pH environment (Fig 12).

### Effect of NKP on EFG-induced signal transduction and actin cytoskeleton machinery

**Fig 13:**
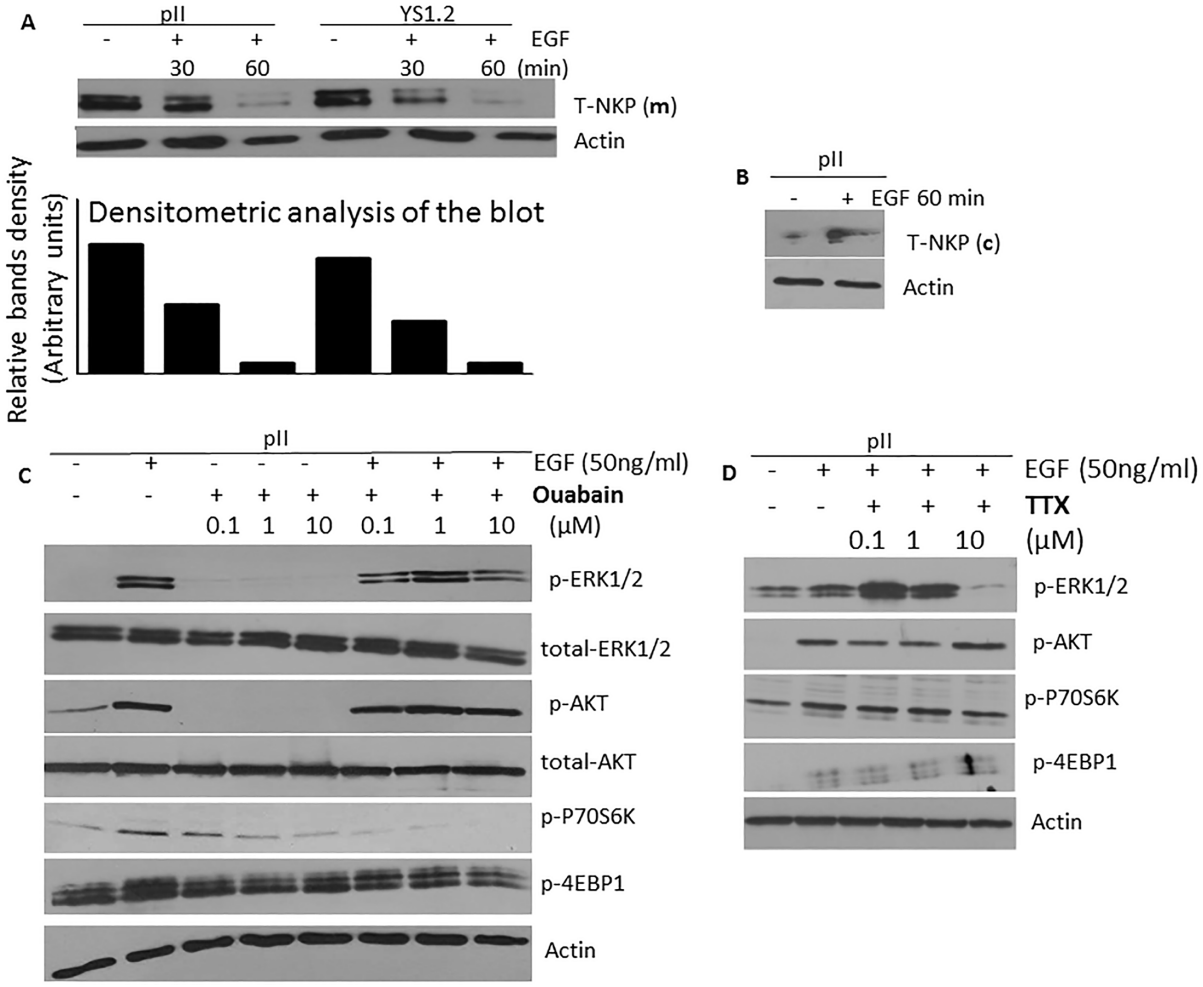
Effect of EGF treatment on NKP localization and the expression/activity of downstream signaling molecules in pII cells. NKP protein expression in the membranous (A) and the cytoplasmic (B) fraction was determined by western blotting in pII cells treated with vehicle or EGF (50 ng/ml, 30–60 min). Protein lysate (3 μg) was electrophoresed on 10% SDS polyacrylamide gel, blotted onto nitrocellulose membrane and probed with antisera to T-NKP and actin (loading control). This blot represents one of 3 experiments. Panels C and D: cells were stimulated with EGF (50 ng/ml for 30 min) in the presence or absence of various concentrations of ouabain or TTX. Total protein lysate (3 μg) was electrophoresed on 10% SDS polyacrylamide gel, blotted onto nitrocellulose membrane and probed with antisera to phospho- or total- ERK 1/2, AKT, P70S6K, 4EBP1, and actin (loading control). This blot represents one of 3 experiments.

Treatment of breast cancer cells with EGF induced the internalization of NKP from the cell membrane (Fig 13A) to the cytoplasmic compartment (Fig 13B). This might suggest a role of NKP in modulating EGF-induced signaling transduction and effector functions. Indeed, western blotting analysis suggests that ouabain treatment reduced EGF-induced phosphorylation of ERK 1/2 and P70S6K (the downstream target of mTOR pathway), but not AKT or 4EBP1 (Fig 13C). On the other hand, TTX treatment only reduced EGF-induced phosphorylation of ERK1/2 (Fig 13D).

**Fig 14:**
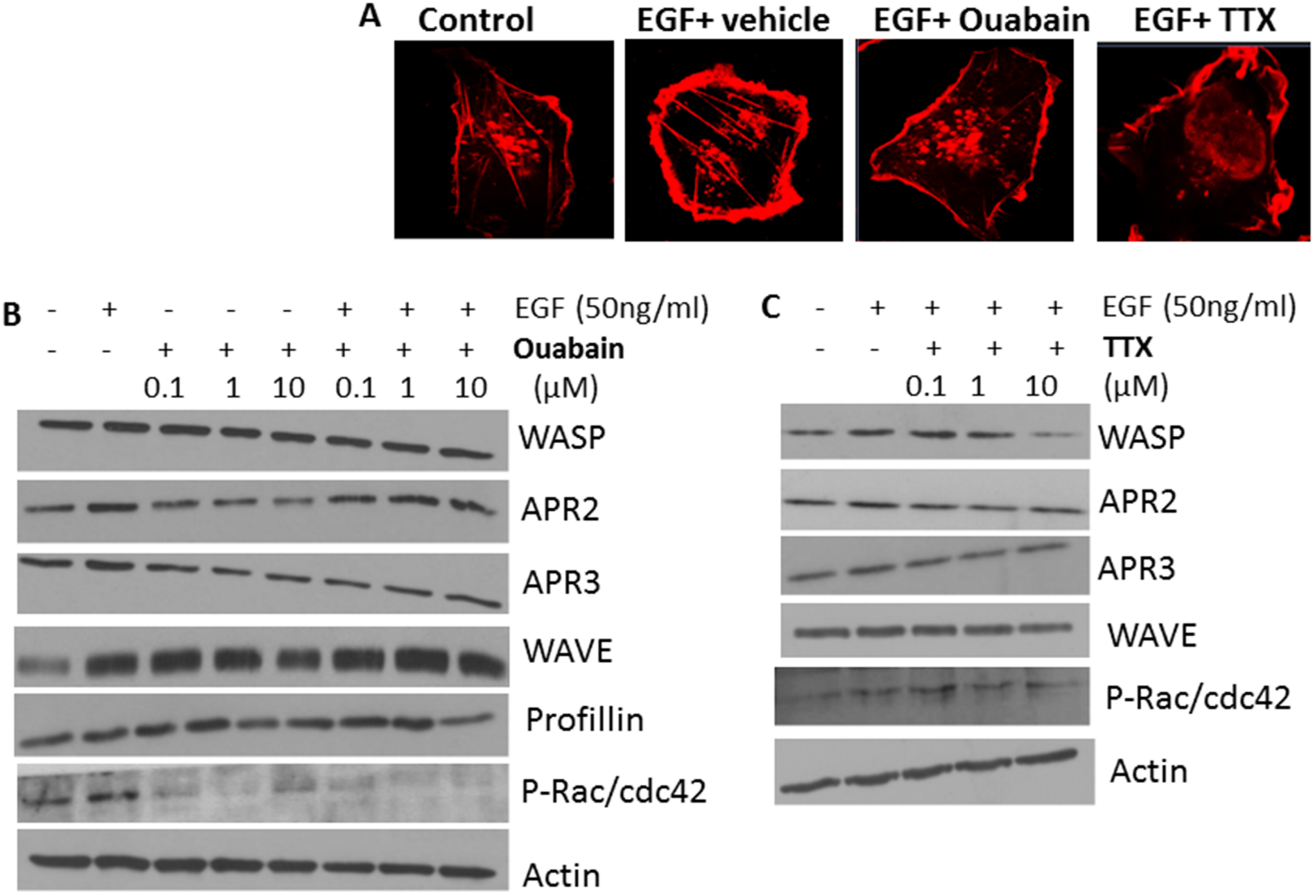
Effect of NKP on EGF-induced membrane ruffling and the expression/activity of actin cytoskeleton-related molecules in pII cells. Panel A: cells were seeded into 8-chambered slides and allowed to grow for 48 h at 37o C/ 5% CO2. Cells were treated with vehicle (control), EGF alone (50 ng/ml, 1 h), or EGF plus ouabain or tetrahydroxyxanthone pre-treatment (1 μM for 1 h), fixed and stained with phalloidin (red). Panels B and C: cells stimulated with EGF (50 ng/ml for 30 min) in the presence or absence of various concentrations of ouabain or TTX. Total protein lysate (3 μg) was electrophoresed on 10% SDS polyacrylamide gel, blotted onto nitrocellulose membrane and probed with antisera to phospho- or total- WASP, APR2, APR3, WAVE, profillin, rac/cdc42, and actin (loading control). This blot represents one of 3 experiments.

Furthermore, immunofluorescence staining of the actin cytoskeleton in pII cells indicated that NKP inhibitors reduced the EGF-induced membrane ruffling needed for cell motility/invasion (Fig 14A). This was in part mediated through reduction in the expression/phosphorylation of key molecules involved in the actin machinery such as profillin and rac/cdc42 by ouabain (but not TTX) treatment (Fig 14B). On the other hand, TTX treatment specifically inhibited the expression of WASP (Fig 14C).

## Discussion

In this study, we have shown that a significant increase in NKP expression/activity was observed in breast cancer cells compared to the non-tumorigenic cells MCF10-A. Inhibiting the NKP activity (using chemical inhibitors or siRNA-mediated knockdown) significantly reduced various effector functions of the highly invasive ER-ve breast cancer cells such as proliferation, motility, blebbing (induced by alkaline pH) and migration/invasion. EGF (which is a known potent stimulus for cancer cell invasion) stimulation induced the internalization of the NKP from the cell membrane to the cytoplasm, and ouabain treatment inhibited EGF-induced membrane ruffling and the activation of ERK1/2 and P70S6K (which are important signaling molecules for many effector functions). Ouabain was generally more potent when compared to TTX in inhibiting various effector functions in pII cells in part through more potent inhibition of MMP activity and increased expression/phosphorylation of P70S6K, profillin and rac/cdc42.

Other studies have reported the anti-proliferative effect of NKP inhibitors on various cancerous and non-cancerous cell lines. Ouabain treatment (1–4 days, at μM concentrations) significantly reduced the proliferation of various prostate cancer cell lines in part through the reduction of mitochondrial activity, increased expression of prostate apoptosis response-4 (par-4) and enhanced production of reactive oxygen species (ROS) [28, 29]. This anti-proliferative effect was also confirmed using the pig kidney epithelial cell line LLC-PK1 [30]. In regard to breast cancer, one report showed that ouabain treatment enhanced the proliferation of the human breast epithelial-derived cancer cell line BT20, as well as the ER-ve breast cancer line MDA-MB-435 [30]. Other reports have demonstrated that ouabain (130–150 nM) treatment inhibited the proliferation of both ER+ (MCF-7) and ER-ve (MDA-MB-231) cells [31]. Kometiani *et al* [19] demonstrated that ouabain (100 nM) significantly reduced MDA-MB-231 cell proliferation, in part through enhanced Src/EGFR activity which leads to ERK1/2 phosphorylation, and reduced expression of p53 and the cell cycle inhibitor p21^cip1^. This is in agreement with our finding in regards to the potent anti-proliferative effect of ouabain (and to a lesser extent TTX) in breast cancer cell lines (Figs 4 and 5), in part through modulation of apoptosis and cell cycle machinery (Fig 6).

In regard to cell motility/invasion, Pongrakhananon *et al* [32] showed that ouabain decreased non-small cell lung cancer cell (NSCLC) motility, migration and invasion, in part through reduced focal adhesion kinase (FAK), ERK and PI3K activation and cdc42 expression. Furthermore, Liu *et al* [33] demonstrated that ouabain significantly reduced basal and EGF-induced migration of the NSCLC cell line A549 through lowering of MMP2/9 activity. These data are in agreement with our findings regarding the potent anti-motile (Figs 7–9) and anti-invasive (Fig 10) properties of the NKP in ER-ve breast cancer cells which were in part through inhibition of MMP activity (Fig 10E). We have recently shown that brief exposure of particularly ER-ve breast cancer cells to alkaline (but not acidic) pH environment results in cell shrinkage, segregation, formation of ring-like bleb structures on the cell`s outer membrane and enhanced invasion toward serum components and EGF, in part through enhanced MMP2/9 activity [26]. We showed that NKP was localized on the outer membrane of the newly formed blebs upon exposure of pII cells to alkaline pH environment (Fig 11D) further confirming the role of this pump in cell invasion. Indeed, ouabain pre-treatment inhibited pII cell shrinkage and membrane blebbing upon subsequent exposure to alkaline pH (Fig 12).

Some evidence suggests the importance of NKP in signal transduction in cardiac myocytes [5, 34], in part through the interaction with EGF receptor to modulate various downstream molecules such as ERK1/2 [9, 35]. EGF is considered an important growth factor in enhancing various effector functions of breast cancer cells [36]. We showed that EGF treatment induced the internalization of NKP from the cell membrane of pII cells to the cytoplasmic compartment and ouabain treatment inhibited EGF induced phosphorylation of p70S6K and ERK 1/2 in agreement with previous reports [9, 35]. These data suggest the importance of ouabain in inhibiting the NKP activity and its downstream signaling effects. It should be noted that the other NKP inhibitor TTX inhibits the pump function (Fig 2D) without modulating the MMP activity (10E) or the activity of the downstream signaling cascade (such as Src complex), P70S6K (Fig 13D), or the actin cytoskeleton-related molecules profillin and rac/cdc42 (Fig 14C, when compared to ouabain treatment). This might explain its reduced potency in inhibiting breast cancer cell proliferation, motility and invasion compared to ouabain.

Although ouabain and TTX treatment (1–10 μM) inhibited NKP activity to similar levels (40–50%, Fig 2D), our data suggest that the effect of these agents on various functions of breast cancer cells may be beyond inhibiting the pump activity. For example, siRNA-mediated knockdown of the NKP-alpha subunit inhibited cell motility by 40% and invasion by 50–80%, compared to 50–60% inhibition of motility and 50–90% inhibition of invasion by ouabain treatment (1–10 μM, Fig 10). TTX treatment was in general less effective than ouabain in inhibiting cell proliferation, motility and invasion in part due to affecting different downstream signaling molecules important for these functions. One clear difference between these two agents is highlighted by the efficacy of ouabain in inhibiting total MMP activity which was not affected by TTX treatment (Fig 10). In addition, ouabain treatment was more potent in inhibiting the expression/activity of ERK1/2, P70S6K, profillin and rac/cdc42 when compared to TTX treatment. Inhibiting these downstream molecules by ouabain suggests that it acts not only as an NKP inhibitor but also affecting other molecules important in breast cancer pathogenesis.

Breast cancer is the second commonest malignant neoplasm and the fifth commonest cause of cancer-related death in women worldwide. Endocrine-based therapies are the treatment of choice for patients with estrogen receptor ER+ tumour cells [37] and have resulted in significant improvements in quality of life and overall survival rates [38]. However, both *de novo* and *acquired* resistance to endocrine-based drugs (such as the anti-estrogen tamoxifen) occurs because of structural or functional loss of the ER [36] and leads to a more aggressive form of disease with poor prognosis. The treatment options for breast cancer patients with ER–ve status are narrowed to the use of chemotherapeutic agents which have serious side effect profile and poor treatment outcomes. Therefore, there is a need to find new therapeutic agents with good efficacy and low side effect profile. Cardiac glycosides (CGs) are a class of compounds which inhibit specifically the activity of the NKP resulting in the intracellular accumulation of Na^+^, which drives the antiporter activity of the Na^+^/Ca^2+^ exchanger (NCX) to promote intracellular accumulation of calcium, which exerts a positive inotropic effect on the cardiac myocytes [39]. They include the cardenolides ouabain and digoxin, and the bufadienolides marinobufagenin, 19-nor bufalin, 3b-hydroxy 14a 20:21-bufenolide, proscillaridin A and telocinobufagin. They have been used to treat patients with heart failure and/or arrhythmia [40] and showed good safety profile. NKP inhibitors have recently obtained more attention due to their strong anti-cancer effects in multiple preclinical assays and have reached early clinical trials [41–44]. In this report, we have provided experimental evidence to support the use of cardiac glycosides (ouabain) for the treatment of breast cancer, especially the endocrine resistant form.

Long-term use of NKP inhibitors such as ouabain should be taken with caution. Some reports have suggested that low doses of ouabain can cause hypertrophy in cardiac myocytes and stimulate the proliferative capacity of smooth muscle and epithelial and endothelial cells [34, 45, 46] and induce cardiac arrest in guinea-pigs [47, 48]. Other reports state that the chronic use of cardiac glycosides is limited by their arrhythmic toxicity. This has been attributed to excessive accumulation of intracellular calcium resulting from inhibition of NKP ion transport activity, and increase in intracellular reactive oxygen species [49]. Also, ouabain had an ototoxic effect *in vivo* and induced spiral ganglion neuron apoptosis *in vitro* in part through the stimulation of the p53 pathway [50]. Furthermore, ouabain treatment in mice was shown to induce cochlear nerve degeneration [51] and glomerular podocytopathies and proteinuria through nephrin downregulation [52]. One report suggested that chronic administration of ouabain in Wistar rats induced hypertension in part through increased Rho kinase activity [53]. Therefore, more studies are needed to assess the long-term safety profile for ouabain as a possible therapeutic approach for breast cancer treatment.

## Materials and methods

### Cell lines

MCF10A non-tumorigenic cells were obtained from Dr. E Saunderson St Bartholomew’s Hospital, London. MCF-7 (ER+) and MDA-MB-231 (ER–ve, *de novo* endocrine resistant) breast cancer cells were obtained from the American Type Culture Collection (VA, USA). pII and YS2.5 (ER-ve, *acquired* endocrine resistant), and YS1.2 (ER+, siRNA transfected but failed to down-regulate ER) cell lines were established in this laboratory by transfection of the original MCF-7 cells with ER directed shRNA plasmid [24, 54]. In brief, complementary oligos (synthesised by Eurogentec, Belgium) containing an ER directed shRNA sequence 5′GCTTCAGGCTACCATTATGttcaagagacataATGGTAGCCTGAAGCttttttacgcgt-3′ (ER sense and anti-sense shown in uppercase and intervening loop sequence and transcription termination sequences shown in lower case) were hybridised, purified and cloned into the Hind III/ Xho1 site of pSingle-tTS vector following the manufacturer’s cloning protocol (Clontech, UK). Standard CaCl_2_ transformation into DH5α E coli was followed by plasmid isolation and purification using Qiagen mini kits (Qiagen, CA, USA). Transfection into MCF7 was performed using TransFast reagent (Promega, Southampton, UK) as per the manufacturer’s protocol. Cell lines SY1.2 and SY2.5 were established from two G418 (1 mg/ml) resistant clones.

For routine culture, cell lines were maintained as monolayers in advanced Dulbecco’s minimum essential medium (DMEM) containing phenol red and supplemented with 5% fetal bovine serum (FBS), 600 μg/ml L-glutamine, 100 U/ml penicillin, 100 μg/ml streptomycin and 6 ml/500 ml 100 x non-essential amino acids (all from Invitrogen, CA, USA), and grown at 37°C in an incubator gassed with an atmosphere of 5% CO_2_ and maintained at 95% humidity. For YS2.5 and YS1.2, the maintenance medium contained G418 (Geneticin, 1mg/ml) but this was omitted during the experiments. MCF10A were maintained as monolayers in DMEM F12 supplemented with 10% horse serum, penicillin/streptomycin, EGF (20 ng/ml), insulin (10 μg/ml), hydrocortisone (0.5 μg/ml) and cholera toxin (100 ng/ml).

### RNA extraction

RNA was extracted from cells and purified using the RNeasy Kit (Qiagen, USA) following the manufacturer’s protocol. The concentration and yield of RNA were determined spectroscopically using the Nano-Drop (Pharmacia) and integrity checked by agarose gel electrophoresis.

### Quantitative real-time PCR

RNA was converted to cDNA using a High-Capacity cDNA Reverse Transcription Kit from Applied Biosystems. Quantitative PCR was performed in the ABI7500 FAST thermocycler in a reaction volume of 20 μl using the SYBER green master mix (from Invitrogen, USA). Primers for *NKP alpha subunit* gene (forward primer 5’-GCTTGAGGCTGTCATCTTCC-3’, reverse primer 5’-TGCGTTTGGCAGTAAGTGTC-3’), and β actin were synthesized in the HSC Research Core Facility, Kuwait University.

### Transient transfection with siRNA against NKP

Cells were seeded into 12-well plates and cultured for 24 h to reach 50–60% confluency before transfection using Stemfect reagent. The procedure was performed according to the manufacturer’s protocol. In brief, solution A (25 μl of stemfect buffer + 2 μl reagent), and solution B (25 μl of stemfect buffer + 20 pmol of siRNA) were prepared and mixed gently and incubated for 15 min at room temperature. For each well, siRNA-stemfect complex was added drop-wise to the wells containing 1 ml DMEM media, and placed in the 37°C, 5% CO_2_ incubator for 48 h. Knockdown of NKP gene was assessed by qPCR following RNA extraction, with transformation of Ct values using the Pfaffl equation.

### NKP activity assay

Cells were seeded into a 25cm^2^ flask and cultured to reach 70% confluency. Cells were left untreated or treated with ouabain/TTX (1–10 μM for 1 h). Cells were then harvested by scraping into 500 μl lysis buffer containing 50 mM HEPES, 50 mM NaCl, 5 mM EDTA, 1% Triton X, 100 μg/ml PMSF, 10 μg/ml aprotinin and 10 μg/ml leupeptin. The lysate was then centrifuged at 1000 rpm for 5 min to pellet cell debris. The supernatant was removed and centrifuged at 41,000 rpm for 40 min. The pellet (membranous fraction) was re-suspended in 1 ml of lysis buffer. The protein concentration was estimated by Bradford assay using BSA as standard. The NKP activity was determined using an ATPase assay kit from Innova Biosciences (Cat# 601–0120) according to the manufacturer’s protocol.

### Immunofluorescence

Cells grown overnight at 37°C, 5% CO_2_ in 8 well glass chambered slides (Lab-Tek, USA) were left untreated (UT, pH 7.4), exposed to acidic (6.3) or alkaline (8.3) pH for 1 h, or stimulated with EGF (50 ng/ml, 30 min). All cells were then fixed with 3.7% paraformaldehyde, washed with 1% BSA and incubated overnight at 4°C with NKP primary antibody (Cell Signaling, USA) diluted 1:50 in 1% BSA. After several washes in PBS, cells were incubated with the secondary antibody (1:500 dilution in 1% BSA, Cell signaling, USA) for 1h in the dark at room temperature. Cells were stained with phalloidin (red stain) to visualize F-actin and DAPI (blue stain) to visualize the nuclei and examined by confocal fluorescence microscopy.

### Apoptosis assay

Cells were cultured in 6 well plates and left untreated (UT, control) or treated with ouabain (100 nM-1 μM) for 24–48 h. The cells were then trypsinized, pelleted by centrifugation at 1000 g for 3 min and washed twice by re-suspension and centrifugation in ice-cold PBS and once in Annexin-V binding buffer (10 mM HEPES/NaOH (pH 7.4), 0.14 M NaCl, 2.5 mM CaCl_2_). The final cell pellet was re-suspended at 5x10^6^ cells/ml in 100 μl Annexin-V binding buffer and processed for FACS analysis using the PE Annexin V apoptosis detection kit I (BD Pharmingen, USA). Cells were stained in the following manner: (A), cells only (negative control) (B), with 10 μl of Annexin V-PE (C), with 20 μl of 7AAD (D), with 10 μl of Annexin V-PE plus 20 μl 7AAD. All incubations were performed in the dark for 15 min at room temperature.

### MTT assay

Approximately 10^4^ cells were seeded into triplicate wells of 12-well plates and allowed to attach overnight. Growth was assessed by MTT assay after 1 or 4 days of incubation with various doses (1 nM-100 μM) of ouabain or 3,4,5,6-tetrahydroxyxanthone (TTX). Control cells received vehicle only. In another experimental set up, cells were treated with estradiol or tamoxifen for 4 days. Briefly, 1 ml of MTT [3-(4,5-dimethyl thiazolyl-2)-2,5-diphenyltetrazolium bromide] reagent (Promega) (0.5 mg/ml) was added to each well and plates incubated at 37°C for 30 min followed by the addition of 1 ml acidic isopropanol and vigorous re-suspension of the converted blue crystals. The absorbance of the suspension was measured at 595 nm with background subtraction at 650 nm. The concentration of either ouabain or TTX that gave half-maximal response (IC_50_) was calculated using non-linear regression analysis by using GraphPad Prism software (version 5.0). The data were fitted to a dose-response-inhibition equation (log [inhibitor] vs. normalized response curve). Results were expressed as mean ± standard error of the mean (S.E.M.)

### Cell cycle analysis

Approximately 10^4^ cells were seeded into triplicate wells of 6 well plates and allowed to attach overnight. The cells were then treated with vehicle (control) or ouabain (100 nM-1 μM) for 24–48 h. The cells were trypsinized and washed with ice-cold PBS. The pellet was then re-suspended in PBS and fixed by adding 70% ethanol. Samples were stored at -20°C overnight. On the following day, samples were centrifuged and washed with PBS. Pellets were then treated with RNase and incubated for 15 min at 37 °C. This was followed by the addition of 200 μl propidium iodide (PI) solution (from 50 μg/ml stock solution). The samples were analyzed using Flow Cytometer Cytomics FC500 with a maximum emission of 605 nm. Data were analysed using CXP Software.

### Motility assay

Cells were cultured in 6 well plates with complete DMEM to 80–90% confluence and treated with vehicle or different concentrations (1 nM-100 μM) of ouabain or TTX. In another experimental setup, cells were transfected with siRNA targeting NKP (48 h) or scrambled vector (control). A scratch was created in the cell monolayer using a sterile p1000 pipette tip and a photograph of the scratched area was taken immediately (0 h), and after 24 h incubation in a 37°C, 5% CO_2_ incubator. The width of the scratch at 24 h was calculated as a percentage of the width at 0 h.

### Agarose migration assay

Ultra-pure agarose (Invitrogen, USA) was melted in PBS, cooled below 40°C, supplemented with complete DMEM and allowed to solidify in individual wells of 6 well dishes at room temperature. Once set, 1–2 sample chambers (3.5 mm in diameter) were created in the gel, 2.5 mm apart in a horizontal line, by insertion of a metallic mould as described previously [25, 26]. Cells (4x10^4^) were re-suspended in complete DMEM containing vehicle (control) or various concentrations (10 nM-10 μM) of ouabain or TTX. In another experimental setup, cells were transfected with siRNA against NKP (48 h) or scrambled vector (control) and then loaded into pre-formed chambers. Plates were then placed at 37°C in the 5% CO_2_ incubator overnight. After 24 h, cells that had penetrated into the agarose were manually counted by visual microscopic examination. Random cell migration towards serum components or EGF (50 ng/ml) was determined as the total number of cells which moved in both lateral directions from the well.

### Matrigel invasion assay

Cells were cultured until reaching 80–90% confluence and starved in serum-free media for 16 h. Basement membrane extract solution (BME) was loaded into the upper well (50 μl/well) and allowed to solidify at 37°C in the 5% CO_2_ incubator overnight. On the following day, cells were harvested and washed twice with PBS, treated with vehicle (control) or ouabain (100 nM-1μM), loaded into the upper well (50,000 cells/well) and incubated at 37°C in the 5% CO_2_ incubator overnight to allow them to invade through the BME into the lower well containing either 150 μl of PBS (control) or serum (10% FBS). On the next day, the lower chamber was rinsed with 1x washing buffer and 100 μl of calcein solution was added (12 μl of calcein AM solution mixed with 1ml of 1x cell dissociation solution) followed by incubation at 37°C in the 5% CO_2_ incubator for 30 min. Light absorbance by the solution in the lower chamber was determined at 485nm excitation, 520 nm emission to determine the number of invading cells by reference to standards.

### Western blotting

Cells were cultured in 6 well plates to 80–90% confluence and treated with vehicle (control) or EGF (50 ng/ml for 30 min) in the presence or absence of different concentrations of ouabain (0.1–10 μM, 30 min pre-treatment). The medium was subsequently aspirated off and cell monolayers harvested by scraping and re-suspension into 300 μl of lysis buffer containing 50 mM HEPES, 50 mM NaCl, 5 mM EDTA, 1% Triton X, 100 μg/ml PMSF, 10 μg/ml aprotinin and 10 μg/ml leupeptin and stored at -80° C. Protein concentration was determined by the Bradford assay using BSA as standard, and 3 μg protein lysate was mixed with an equal volume of 2 x SDS and heated at 90°C for 10 min. Samples were loaded onto a 10% SDS-polyacrylamide gel and electrophoresed at 150 V for 1 h. Proteins were transferred to a nitrocellulose membrane and blocked with 2% BSA for 1 h before being incubated overnight at 4°C with primary antibodies (prepared in 2% BSA) against actin (loading control, 1:1000 dilution), phospho- or total- NKP, ERK 1/2, AKT, P70S6K, 4EBP1, WASP, APR2 and 3, WAVE, profillin, and Rac/cdc42 (all from Cell Signaling, USA, 1:100 dilution). In another experimental setup, the membranous fraction of cells was isolated and lysed, and NKP expression was determined using primary antibody against NKP (Cell Signaling, USA, 1:100 dilution). The membrane was then washed and incubated with anti-HRP-conjugated secondary antibody (1/500 dilution) for 1 h (Cell Signaling, USA), developed with Super Signal ECL and visualized with Kodak X-ray film.

### Matrix metalloproteinase activity

The general activity of MMPs was determined using a kit from Abcam (cat no. ab112146) as previously described [27].

### Statistical analysis

Student’s two-tailed unpaired t-test, or one-way ANOVA test followed by Bonferroni post hoc test were used to compare means of individual groups: p < 0.05 was considered statistically significant.
